# Multidrug resistant tuberculosis in prisons located in former Soviet countries: A systematic review

**DOI:** 10.1371/journal.pone.0174373

**Published:** 2017-03-23

**Authors:** Maxwell Droznin, Allen Johnson, Asal Mohamadi Johnson

**Affiliations:** 1 Department of Health Professions, Rollins College, Winter Park, Florida, United States of America; 2 Department of Sociology, Anthropology, and Public Health, Stetson University, DeLand, Florida, United States of America; University of Padova, Medical School, ITALY

## Abstract

**Background:**

A systematic literature review was performed to investigate the occurrence of multidrug-resistant tuberculosis (MDR TB) in prisons located in countries formerly part of the Soviet Union.

**Methods:**

A systematic search of published studies reporting MDR TB occurrence in prisons located in former Soviet countries was conducted by probing PubMed and Cumulative Index Nursing and Allied Health Literature for articles that met predetermined inclusion criteria.

**Results:**

Seventeen studies were identified for systematic review. Studies were conducted in six different countries. Overall, prevalence of MDR TB among prisoners varied greatly between studies. Our findings suggest a high prevalence of MDR TB in prisons of Post-Soviet states with percentages as high as 16 times more than the worldwide prevalence estimated by the WHO in 2014.

**Conclusion:**

All studies suggested a high prevalence of MDR TB in prison populations in Post-Soviet states.

## Introduction

It is estimated that as of 2014, there were 480,000 cases of MDR TB worldwide [1]. In 2015, of the 27 countries worldwide designated as having a high burden for multidrug-resistant tuberculosis (MDR TB) by the World Health Organization (WHO), 15 of them were in Post-Soviet states [2]. In fact, of all MDR TB cases reported in 26 European countries between 2003 and 2007, 65% were concentrated in three Post-Soviet states: Latvia, Lithuania and Estonia [3]. MDR TB takes longer to diagnose, longer to treat, costs more money and has a greater risk of treatment failure than drug-susceptible tuberculosis [4–6]. While MDR TB can arise when patients do not complete a full course of anti-TB medications, it can also be transmitted from person-to-person via infected droplets in the air, even to those who have never taken anti-TB drugs [7–10]. This makes MDR TB a particular concern in crowded, in-door areas, such as prisons.

Prisoners, on average, have a higher prevalence of tuberculosis (TB) relative to the general population [11,12]. This can be explained in part due to the fact that prisoners often come from populations that are considered high risk for developing TB (substance abuse, mental illness, homelessness). However, prisons themselves can facilitate the spread of TB via poor ventilation systems, overcrowding and poor nutrition [13–17]. Not only does this represent a threat to prisoners, but to the general population as a whole. Prisoners who acquire TB can spread it beyond the confines of their prison cells through daily interaction with prison staff, visitors and prisoners who are about to be released [18].

Post-Soviet states have some of the highest incarceration rates in the world [19], and the TB epidemic within those prisons is well documented [20]. Mistreatment of prisoners in former Soviet states continues to be a major issue and may suggest negative Soviet era attitudes towards prisoners persist [20,21]. In addition to economic and health system collapse following the dissolution of the Soviet Union, these stigmas could also be contributing to inadequate attention to prisoner health.

Despite being more than half of the world’s high burden MDR TB countries, there has never been a direct comparison of the literature on MDR TB in prisons of the Post-Soviet states [2]. While studies exist that report global MDR TB, they fail to elucidate the scope of the problem in the Post-Soviet region. Thus, the aim of this study is to provide the first systematic review of studies that examine the prevalence of MDR TB in Post-Soviet prisons.

## Methods

### Definition of Post-Soviet states

Also known as the former Soviet Union, Post-Soviet states refers to the 15 independent nation-states that emerged following the collapse of the Union of Soviet Socialist Republics (USSR) in 1991. These include Armenia, Azerbaijan, Belarus, Estonia, Georgia, Kazakhstan, Kyrgyzstan, Latvia, Lithuania, Moldova, Russia, Tajikistan, Turkmenistan, Ukraine, and Uzbekistan [22,23].

### Definition of multidrug-resistant tuberculosis

MDR TB is defined by the WHO as being resistant to isoniazid and rifampicin, with or without resistance to any other first-line drugs [24]. All studies included in this review followed that definition when reporting MDR TB cases [25–41].

### Literature search strategy and inclusion criteria

This study was not registered with any systematic review protocol registry. Because the Soviet Union dissolved in 1991, we included articles published between 1992 and 2015. PubMed and Cumulative Index Nursing and Allied Health Literature (CINAHL) were searched for relevant studies based on predetermined inclusion criteria. The following MeSH terms were used: tuberculosis, TB, multi drug-resistant, prison, jail, incarcerated, inmate, penitentiary, and the individual names of all the countries that are defined as Post-Soviet. Studies were included if they were original research, published in a peer-reviewed scientific journal, were in the English language, focused on one or more Post-Soviet countries, included prisoners as the study population, reported prevalence of MDR TB among prisoners, and used data that were collected between 1992 and 2015.

**Fig 1:**
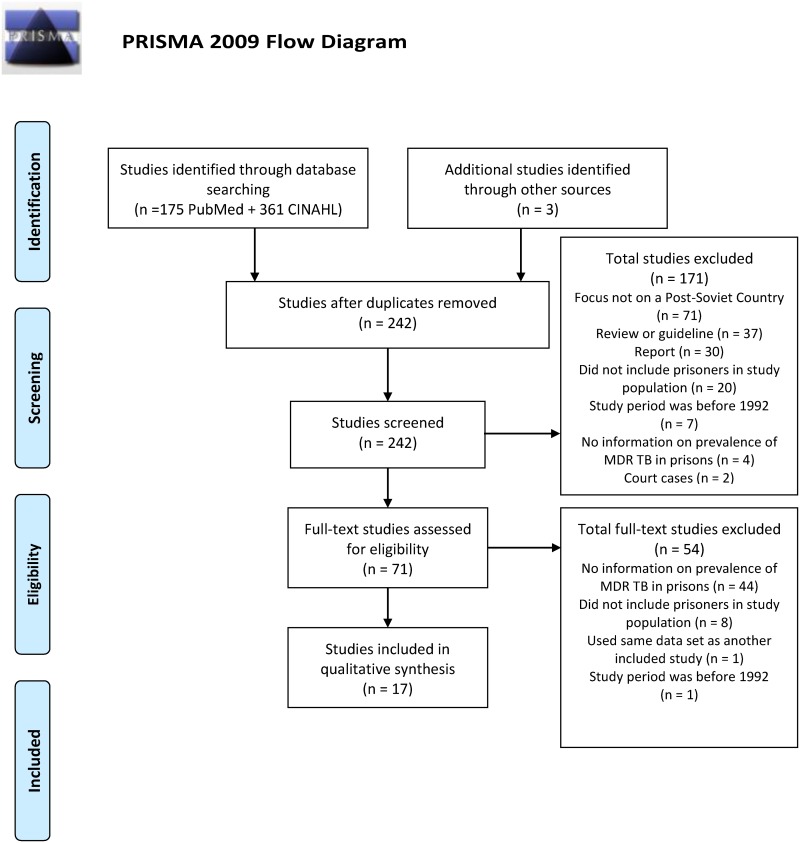
PRISMA 2009 flow diagram. *From*: Moher D, Liberati A, Tetzlaff J, Altman DG, The PRISMA Group (2009). *P*referred *R*eporting *I*tems for Systematic Reviews and *M*eta-*A*nalyses: The PRISMA Statement. PLoS Med 6(7): e1000097. doi: 10.1371/journal.pmed1000097. **For more information, visit** www.prisma-statement.org.

A total of 239 potentially eligible studies were initially identified. We subsequently excluded 225 studies for the following reasons: 71 did not focus on a Post-Soviet country, 48 did not have information on MDR TB prevalence in prisons 37 were reviews or guidelines, 30 were reports, 28 did not include prisoners in the study population, 8 included a study period before 1992, 2 were excluded because they were court cases, and 1 was excluded because it used the same data set as a previously included study. Three additional studies that matched all of the inclusion and none of the exclusion criteria were brought in through looking at sources of other review papers. Seventeen studies were included for review. Two researchers (AJ and AMJ) independently assessed the methodological integrity of each included study. Fig 1 provides a flow diagram of the inclusion and exclusion process.

## Results

A total of 17 studies met the inclusion criteria for systematic review [25–41]. Characteristics of included studies are presented in Table 1. Seven of the studies reported MDR TB in both prisoner and civilian populations [26,30–33,35,36]; whereas, 10 of the studies only reported MDR TB among prisoners [25,27–29,34,37–41]. The majority of studies were cross-sectional studies [25–29,32–41] and two were prospective cohorts studies [30,31]. Five of the studies used data from a national database [25,29,32–34], while, 12 used regional data [26–28,30,31,35–41]. Ten studies were conducted in Russia [26–28,30,31,35,37,38,40,41], 3 in Georgia [25,33,34], 1 in Moldova [32],1 in Azerbaijan [29], 1 in Kazakhstan [36], and 1 in Kyrgyzstan [39]. The studies done in Russia, Kazakhstan and Kyrgyzstan used regional data; whereas, studies in Georgia, Moldova and Azerbaijan were national studies [25–41].

**Table 1:** Characteristics of included studies.

Author, Year [Citation]	Country	Study Design	Data Sources	Participants
Aerts, 2000 [25]	Georgia	Cross-sectional	Prison system of Georgia between 1997 and 1998	445 TB positive prisoners
Balabnova, 2006 [30]	Russia	Prospective cohort	Patients observed in Samara Oblast, Russia between 2002 and 2008	2099 TB positive patients (640 prisoners and 1459 civilians)
Balabnova, 2011 [31]	Russia	Prospective cohort	Patients recruited in Samara Oblast, Russia between 2002–2003	880 TB positive patients (164 prisoners, 716 civilians)
Bonnet, 2005 [37]	Russia	Cross-sectional	Kemerovo prison, Kemerovo Oblast, Russia in 2001	459 TB positive prisoners
Ibrayeva, 2014 [36]	Kazakhstan	Cross-sectional	Prison systems of Karaganda and Akmola regions in 2012 “Civilian sector” of Kazakhstan	185 TB positive patients (60 prisoners, 125 civilians)
Ignatova, 2006 [28]	Russia	Cross-sectional	Ozerki prison hospital, Tula Oblast, Russia between June, 2001 and June, 2002	87 TB positive prisoners
Jenkins, 2013 [32]	Moldova	Cross-sectional	Moldovan TB database of all notified TB cases diagnosed nationwide between January 2007 and December 2010	23,152 TB positive patients (9,071 testable cases with 606 prisoners and 8,465 civilians)
Jenkins, 2014 [33]	Georgia	Cross-sectional	All notified TB cases in Georgia between 2009 and 2011 All patients hospitalized and started on SLD in Georgia between 2009 and 2011	6931 TB positive patients (1,582 prisoners, 5,349 civilians)
Jugheli, 2008 [34]	Georgia	Cross-sectional	Prison system of Georgia between 2001 and 2003	270 TB positive prisoners
Kimerling, 1999 [38]	Russia	Cross-sectional	Colony 33, Kemerovo Oblast, Russia between December 1997 and March 1998	164 TB positive prisoners
Mar'iandyshev, 2005 [41]	Russia	Cross-sectional	Arkhangelsk prison system, Arkhangelsk Oblast, Russia	343 TB positive prisoners
Mokrousov, 2009 [39]	Kyrgyzstan	Cross-sectional	Moldovanovka prison TB hospital, Bishkek, Kyrgyzstan between August and November of 2008	56 TB positive prisoners
Pfyffer, 2001 [29]	Azerbaijan	Cross-sectional	Central Penitentiary Hospital of Azerbaijan	65 TB positive prisoners
Ruddy, 2005 [26]	Russia	Cross-sectional	Samara Oblast, Russia	600 TB positive patients (291 prisoners, 309 civilians)
Shemyakin, 2004 [40]	Russia	Cross-sectional	Serpukhov prison, Moscow Oblast, Russia between January and December 2001	130 TB positive prisoners
Spradling, 2002 [35]	Russia	Cross-sectional	DST records of the Central Tuberculosis Dispensary of Oryol Oblast, Russia from 1 July 1999 through 30 June 2000	212 TB positive patients (41 prisoners, 171 civilians)
Toungoussova, 2003 [27]	Russia	Cross-sectional	Archangel (Arkhangelsk) prison, Arkhangelsk Oblast, Russia	114 TB positive prisoners

All studies reported prevalence of MDR TB among prisoners who were culture positive for TB. Not all prisoners who had active TB were culture positive [25–41], with some studies reporting less than half of all sputum samples being successfully cultured in a laboratory [30,34]. As drug susceptibility testing can only be done with culture positive cases, the actual number of MDR TB cases is likely underestimated.

Overall, prevalence of MDR TB among prisoners varied greatly between studies. One study in the Oryol region of Russia found MDR TB prevalence among prisoners with active TB to be 12.0%; whereas, another study in the Tula region of Russia found a prevalence of 71.2% [28,35]. Since no national MDR TB data exists for Russia, the mean average of MDR TB prevalence data from the Russian studies was used to calculate a national average prevalence among prisoners of 43.14% [26–28,30,31,35,37,38,40,41]. Prevalence in Georgia ranged from 13.0% to 18.1% [25,33,34]. Prevalence of MDR TB among prisoners in Azerbaijan was 52.3%; whereas, in Moldova, it was 65.8% [29,32]. In Kazakhstan, the prevalence of MDR TB in prisons was 81.67%; while, in Kyrgyzstan it was found to be 26.8% [36,39]. In all but one study [29], previously treated TB cases had a higher prevalence of MDR TB than new TB cases [25–28,30–34,36,37,41].

Five studies reported adjusted odds ratios (AOR) using multivariate analysis [25,26,30,33,37]. While one study in Russia reported that those with MDR TB had a 4 times greater odds of being a prisoner [30], a study done in Georgia found no association between MDR TB and prisoners [33]. The same study in Georgia found that older age was associated with decreased odds of MDR TB, while another study in Georgia found the odds of having MDR TB increased with age [25,33].

Prisoners with MDR TB had greater odds of being overweight or obese (body mass index [BMI] > 25kg/m^2^) compared to those with drug-susceptible TB. However, the same study found individuals with drug-susceptible TB were at decreased odds of being overweight or obese. No association between MDR TB and a BMI under 20 kg/m^2^ was reported, but prisoners with drug-susceptible TB had increased odds of having a BMI under 20 kg/m^2^ compared to those with MDR TB [25].

The only study that examined recreational drug users found an association between illicit drug use and MDR TB. However, those with MDR TB were at decreased odds of having HIV [26].

Every study that looked at current or previous TB treatment as a risk factor found significant associations between treatment and MDR TB. Two studies found that those with MDR TB had 2.7 times greater odds of having had previous treatment [25,26]. Another 2 studies found that those with MDR TB had over 5 times greater odds of having had treatment failure [30,37]. One study that followed a cohort of 18 prisoners through TB treatment found that 94% (17) developed MDR TB [38]. The major findings for each study are presented in Table 2.

**Table 2:** Summary of study finding.

Author, Year [Citation]	Prevalence of MDR TB among Prisoners with TB	Other Findings	Adjusted Odds Ratio (AOR [CI^a^])
Aerts, 2000 [25]	13.0% (36 cases out of 276)	Those with MDR TB had a 11.0 times greater odds of having a BMI over 25kg/m^2 compared to those with drug susceptible TB. Those with MDR TB had 0.9 times decreased odds of spending 2 to 4 years in prison. Those with MDR TB had increased odds of being older compared to those with drug-susceptible TB. The proportion of new and previously treated MDR TB were 4 (5.6%) and 32 (15.7%), respectively.	Those who have been in prison 2 to 4 years had decreased odds of MDR TB compared to those who have been in prison either less than 2 years or more than 4 years (AOR = 0.1 [0.0–0.6]). Those who have been in prison for more than 4 years had decreased odds of MDR TB compared to those who have been in prison for less than 4 years (AOR = 0.4 [0.1–1.8]). Those with BMIs greater than 25 had increased odds of MDR TB compared to those with BMIs less than 25 (AOR = 11.0 [1.1–114.7]). Those with BMIs less than 20 had increased odds of MDR TB compared to BMI greater than 20 (AOR = 2.2 [0.8–6.0]). Those receiving previous TB treatment were at increased odds of MDR TB compared to those with no previous TB treatment (AOR = 2.7 [0.7–11.0]). 25–29 years olds had increase odds of MDR TB compared to age groups younger than 25–29 (AOR = 6.8 [1.1–41.1]). 30–39 years olds has increased odds of MDR TB compared to age groups younger than 30–39 (AOR = 6.5 [1.2–35.9]). Those older than 39 years old had increased odds of MDR TB compared to age groups younger than 39 years old (AOR = 9.9 [1.5–66.1]).
Balabnova, 2006 [30]	41.6% (84 cases out of 202 cases)	Among civilians, 12.4% (95) of new cases and 22.4% (17) of previously treated cases were MDR TB. Among the study population, those with MDR TB had 4.4 times greater odds of being a prisoner compared to those who with drug susceptible TB. Among the study population, those with MDR TB had 3.5 times greater odds of having a relapse compared to those with drug susceptible TB. Among the study population, those with MDR TB had 5.0 times greater odds of having unsuccessful treatment compared to those with drug susceptible TB.	Being a prisoner was associated with increased odds of MDR TB compared to being a civilian (AOR = 4.4 [2.7–7.1]). Having a relapse of TB was associated with increased odds of MDR TB compared to being a new case (AOR = 3.5 [1.7–7.1]). Unsuccessful outcomes of treatment was associated with increased odds of MDR TB compared to no previous treatment (AOR = 5.0 [1.1–22.7]).
Balabnova, 2011 [31]	49.3% (33 cases out of 67)	14.8% of civilian TB cases were MDR TB. Among civilians, 13.9% (122) of all new cases and 22.7% (17) of previously treated cases were MDR TB.	Not reported
Bonnet, 2005 [37]	27.9% (128 cases out of 459)	20.8% (60) of new cases were MDR TB and 40.0% (68) previously treated cases were MDR TB.	Treatment failure cases were associated with increased odds of MDR TB compared to cases who had no previous treatment (AOR = 5.3 [3.1–9.2]).
Ibrayeva, 2014 [36]	81.7% (49 cases out of 60)	76.0% (95) of civilian cases were MDR TB. 57.1% (8) of new prison cases were MDR TB and 89.1% (41) of previously treated prison cases were MDR TB. 59.3% (32) of new civilian cases were MDR TB and 88.7% (63) of previously treated civilian cases were MDR TB.	Not reported
Ignatova, 2006 [28]	71.3% (62 cases out of 87)	37.5% (9) of new cases and 84.1% (53) of previously treated cases were MDR TB.	Not reported
Jenkins, 2013 [32]	65.8% (399 cases out of 606)	Estimated total incidence of MDR TB among prisoners was 1,000/100,000 compared to 52/100,000 in general population. Among the general population, 3,447 (38.0%) were MDR TB. 23.5% (1,279) of new cases were MDR TB and 61.5% (2146) of previously treated cases were MDR TB. In prisons, 65.8% (399) of TB cases were MDR TB with 36.9% (83) of new cases and 82.8% (314) of previously treated cases being MDR TB.	Not reported
Jenkins, 2014 [33]	18.1% (287 cases out of 1582)	Total incidence of MDR TB among prisoners was 837.1/100,000 compared to 16.2/100,000 in general population. 10.2% of all new TB cases (538) and 32.7% (521) of all previously treated cases were MDR TB. Prevalence of MDR TB in the general population was 15.5% (1,075). Among the study population, those with MDR TB had 0.28 times lower odds of being over 35 years old compared to those with drug susceptible TB.	Being a prisoner was associated with increased odds of MDR TB compared to being a civilian (AOR = 1.2 [0.9–1.5]). Living in urban areas was associated with increased odds of MDR TB compared to living in rural areas (AOR = 1.4 [1.2–1.7]). Being over 35 years old was associated with decreased odds of MDR TB compared to being younger than 35 years old (AOR = 0.7 [0.6–0.8]).
Jugheli, 2008 [34]	14.4% (39 cases out of 271)	30.8% (12) of MDR TB cases were new cases and 69.2% (27) were previously treated cases.	Not reported
Kimerling, 1999 [38]	22.6% (37 cases out of 164)	After prolonged treatment, 94.0% of patients in an 18 person cohort acquired MDR TB.	Not reported
Mar'iandyshev, 2005 [41]	53.7% (87 cases out of 162)	27.6% (26) of new cases and 89.7% (61) of previously treated cases were MDR TB.	Not reported
Mokrousov, 2009 [39]	26.8% (15 cases out of 56)	All cases were HIV negative.	Not reported
Pfyffer, 2001 [29]	52.3% (34 cases out of 65)	54.1% (33) of new cases and 25.0% (1) of previously treated cases were MDR TB.	Not reported
Ruddy, 2005 [26]	49.8% (145 cases out of 291)	37.3% (25) of all new prison cases and 53.6% (120) of all previously treated prison cased were MDR TB. 19.8% (19) of civilian new cases and 55.6% (119) of previously treated civilian cases were MDR TB. The attributive risk of developing MDR TB in prisoners was 17.5% (95% CI 2.2 to 32.8). Prisoners were nearly twice as likely to have MDR TB compared to the general population (Risk Ratio = 1.9 [95% CI 1.1 to 3.2]). Among the study population, those with MDR TB had 0.7 times lower odds of having an HIV infection compared to those who had drug susceptible TB. Among the study population, those with MDR TB had 2.8 times greater odds of currently taking medications for TB compared to those with drug susceptible TB. Among the study population, those with MDR TB had 3.0 times greater odds of being recreational drug users compared to those with drug susceptible TB. Among prisoners, those with MDR TB had 2.6 times greater odds of having fibrocavity TB compared to those with drug susceptible TB.	HIV positive cases had decreased odds of MDR TB compared to HIV negative cases (AOR = 0.3 [0.1–0.8]). Having fibrocavity TB was associated with increased odds of MDR TB compared to other forms of TB (AOR = 2.6 [1.1–6.9]). New cases that used recreational drugs were at increased odds of MDR TB compared to new cases that did not (AOR = 3.0 [1.1–7.9]). Those receiving TB treatment had greater odds of MDR TB compared to those who were not on treatment (AOR = 2.8 [1.2–6.5]).
Shemyakin, 2004 [40]	65.4% (85 cases out of 130)	All cases were HIV negative.	Not reported
Spradling, 2002 [35]	12.2% (5 cases out of 41)	12.0% of prisoners had MDR TB as compared to 5.0% of civilians. 4.0% of new cases and 32.0% of previously treated cases were MDR TB.	Not reported
Toungoussova, 2003 [27]	37.7% (43 cases out of 114)	The proportion of MDR TB among new and previously treated cases were 34.0% (32) and 55.0% (11), respectively. None of the 114 TB cases tested positive for HIV.	Not reported

^a^Confidence Interval of 95%

## Discussion

The goal of this study was to investigate the prevalence of MDR TB among prisoners of Post-Soviet states. We used systematic review to analyze studies and extrapolate results. Our findings suggest a high prevalence of multidrug-resistance in prisons of Post-Soviet states with percentages as high as 16 times higher than the worldwide prevalence estimated by the WHO in 2014 [1,36]. While there were no common risk factors among studies reporting adjusted odds ratios [25,26,30,33,37], all studies suggested a high prevalence of MDR TB in prison populations in Post-Soviet states.

Within the Soviet Union, even after the advent of antibiotics for TB, the standard procedures for treatment were diagnosis by X-rays, admittance to a sanitarium, isolation, and occasionally surgery [42]. This treatment was, in fact, effective as TB mortality rates in the Soviet Union decreased from 400 per 100,000 persons in the 1910s to 17.3 per 100,000 men and 1.9 per 100,000 women in 1990 [43,44].

In 1991, the Soviet Union dissolved and the entire interconnected health, economic, and prison system collapsed with it. Suddenly millions of people found themselves living in poverty in newly formed nations [45]. Between 1991 and 1998, TB incidence in most Post-Soviet states more than doubled [46]. Additionally, during this time period, both crime and incarceration rates increased in the region as arrests tripled between 1988 and 1995 [20]. The *gulag* prison work camps of the former Soviet Union were repurposed to serve as prisons for the newly formed counties in which they were located. These prisons were not only over-crowded but also characterized by inadequate ventilation and malnutrition [20]. Such conditions resulted in high transmission of TB [7–10]. Between 1991 and 1997 the mortality rate in Russian prisons more than doubled with approximately half of these deaths being attributed to TB [47] and by 1999, one-third of all TB cases in Russia were in prisons [48].

Following the collapse in 1991, many of the newly formed nations started to implement the direct observed therapy short course (DOTS) system as recommended by the WHO. However, among physicians of the Post-Soviet states there was strong opposition to this move as they felt it threatened their livelihoods [49]. Conflicting instructions on how to best treat TB during the early 1990s likely did little to help the situation as isoniazid, rifampin and other TB medications started to enter the market. Partly because of stigmas associated with undergoing DOTS, TB medications took on value and were sold in open air markets, without prescription from pharmacists trying to make ends meet, and traded among inmates in prisons [49,50].

By 1998, it was estimated 20% of all TB cases in Russian prisons were multidrug-resistant [51]. Our findings suggest the prevalence of MDR TB in Russian prisons has since increased, and in some cases, more than doubled [26–28,30,31,35,37,38,40,41]. In Azerbaijan, Moldova, and Kazakhstan the proportion of TB among prisoners that was multidrug-resistant was found to be around 52%, 65% and 81% respectively [29,32,36]. Georgia and Kyrgyzstan, although similar in terms of incarceration rates to Azerbaijan, Moldova and Kazakhstan, had the lowest prevalence of multidrug-resistance with a reported prison prevalence of 18.1% and 26.8%, respectively [19,33,39].

Although, still around triple the global rate estimated by the WHO in 2014 [1], the prevalence rate of MDR TB in Georgian prisons seems to be one of the lowest in the Post-Soviet region [25–41]. To explain the differences in prevalence rates among studies, we looked at several factors including incarceration rates, use of fixed-dose combinations (FDC), nutrition, and per capita gross domestic product (GDP).

Among the countries in this study, lower incarceration rates were not associated with lower MDR TB. The studies from Russia, which had one of the highest rates of incarceration in the world at the beginning of each study period, had a more than 20% lower mean prevalence than Moldova which had one of the lowest rates of incarceration among all the countries in this study [19,26–28,30–32,35,37,38,40,41], Georgia, which had one of the highest rates of incarceration and one of the highest percentages of pre-trial detention in the world [19], had the lowest prevalence of MDR TB in prisons of Post-Soviet states [25,33,34].

The use of FDC has been suggested to account for low MDR TB prevalence in Sub-Saharan Africa [52,53]. However, no information on FDC usage was mentioned in any of the studies [25–41]. Only one study suggested the implementation of FDC as a possible solution for decreasing MDR TB prevalence rate in the Samara Oblast of Russia.^26^

Data on nutrition and daily caloric intake of prisoners in Post-Soviet states has not been found, however several studies have suggested that nutritional status is fairly standard among prisoners in much of the Post-Soviet region [20,21]. There also seems to be conflicting data regarding prison capacity and overcrowding, making it difficult to assess these findings [19,54].

Earlier studies have suggested MDR TB is inversely associated with GDP [55,56]. Our findings, however, did not show such a relationship. Regions with some of the highest GDPs also had the highest rates of MDR TB; whereas, regions with some of the lowest GDPs also had the lowest rates of MDR TB [25–41,57,58]. In fact, among the 3 Georgian studies conducted in 1997, 2001 and 2009, MDR TB seems to be increasing during this period even though during the same period the GDP almost tripled [25,33,34,58]. This may result from better detection and reporting in Post-Soviet states [59,60]. However, Georgia, in comparison to other Post-Soviet states included for review, has a relatively low rate of multidrug-resistance. Further investigation is needed to elucidate possible reasons why.

Since four studies [25,26,30,37] found MDR TB to be associated with TB treatment and the majority of MDR TB cases were from patients who had previously undergone TB treatment [25–28,30–37,41], inadequate or inappropriate treatment and medical noncompliance is likely a mode of spreading MDR TB in Post-Soviet states’ penal systems.

It is important for future research to focus on risk factors so that this epidemic could be better understood and addressed. With conflicting data and small sample sizes of publications, more studies are needed to create a more comprehensive landscape of MDR TB in Post-Soviet prisons. Notably, none of the studies reported a previous history of TB infection among prisoners. Many included previously treated cases, and indeed most of these did have MDR TB [25–28,30–37,41], but studies failed to differentiate between treatment failure cases and those that successfully completed treatment and then were re-infected. Information on the length of prison terms were lacking. Only one study looked at this potential association and found that prisoners who had MDR TB were at decreased odds of having spent a prison term between 2–4 years [25]. This may suggest prison terms less than 2 years and more than 4 years could correlate with MDR TB but further investigation is needed.

Malnutrition has been considered a problem in Post-Soviet prisons [20,21], but only one study included BMI and it found that being overweight was associated with MDR TB [25]. While being overweight is not an indicator of proper nutrition, this link needs to be investigated further. It is not clear if diets varying among countries could have an effect as nutrition has been shown to be a correlated with TB [13–17].

While the link between TB and HIV in prisons has been well established [58–63], the link between MDR TB and HIV is less clear. Studies have found conflicting results and one review focusing on prisons worldwide found that overall there was no significant association, although the relationship was variable based on region [64,65]. The HIV epidemic in the Post-Soviet states has been fueled by injection drug use [62,66,67]. Injection drug use has also been speculated to be associated with increased likelihood of becoming a prisoner as well as acquiring MDR TB [68], but only one of our studies looked at it as a factor [26]. It found that injection drug use was associated with MDR TB but also that those with MDR TB were at decreased odds of having HIV compared to those without MDR TB [26]. The other three studies we included in the review that looked at HIV in prisons found that all MDR TB patients were HIV negative [27,39,40]. As the current sample size of studies is too small to suggest an association, more studies are needed to examine the pathway between injection drug use, MDR TB, and HIV in Post-Soviet prisons.

As suggested by our studies, the rate of MDR TB in prisons of the former Soviet Union is above the world average [1,25–41]. Additionally, consecutive studies done within the same country at different times suggest an increase in MDR TB prevalence overtime [25–27,30,31,33,34,37,38,41]. For effective control, targeted MDR TB control programs tailored to Post-Soviet states, specifically within the penal systems, are warranted.

### Limitations

Our review did encounter some limitations. Because our inclusion criteria included studies published from 1992 to 2015, studies published after this timeframe would not have been identified. Additionally, although PubMed and CINAHL are extensive databases for health research, limiting our search to these two databases may have resulted in the exclusion of any potential studies not cataloged in either PubMed or CINAHIL.
